# Approaching 80 years of snow water equivalent information by merging different data streams

**DOI:** 10.1038/s41597-020-00649-1

**Published:** 2020-10-06

**Authors:** Laurie S. Huning, Amir AghaKouchak

**Affiliations:** 1 Department of Civil Engineering and Construction Engineering Management, California State University, Long Beach, Long Beach, CA 90840 USA; 2 Department of Civil and Environmental Engineering, University of California, Irvine, Irvine, CA 92697 USA; 3 Department of Earth System Science, University of California, Irvine, Irvine, CA 92697 USA

**Keywords:** Cryospheric science, Water resources, Hydrology

## Abstract

Merging multiple data streams together can improve the overall length of record and achieve the number of observations required for robust statistical analysis. We merge complementary information from different data streams with a regression-based approach to estimate the 1 April snow water equivalent (SWE) volume over Sierra Nevada, USA. We more than double the length of available data-driven SWE volume records by leveraging *in-situ* snow depth observations from longer-length snow course records and SWE volumes from a shorter-length snow reanalysis. With the resulting data-driven merged time series (1940–2018), we conduct frequency analysis to estimate return periods and associated uncertainty, which can inform decisions about the water supply, drought response, and flood control. We show that the shorter (~30-year) reanalysis results in an underestimation of the 100-year return period by ~25 years (relative to the ~80-year merged dataset). Drought and flood risk and water resources planning can be substantially affected if return periods of SWE, which are closely related to potential flooding in spring and water availability in summer, are misrepresented.

## Abstract (summary)

**Table:** 

Measurement(s)	snowpack water volume • volume of hydrological precipitation
Technology Type(s)	Statistical Modeling • digital curation
Factor Type(s)	time series
Sample Characteristic - Environment	snowpack • cold environment • hydrological precipitation process
Sample Characteristic - Location	Sierra Nevada

Machine-accessible metadata file describing the reported data: 10.6084/m9.figshare.12902354

## Background & Summary

Merging a variety of data streams together (e.g., remote sensing products and *in-situ* observations) is a valuable technique for hydrologic estimation^1–4^. When complementary information is leveraged, different data streams can be fused together to develop a longer dataset for statistical analysis. Statistical approaches, such as hydrologic frequency analysis, necessitate a sufficient number of observations to estimate representative return periods^5,6^. Drought and flood risk and water resources planning can be substantially affected if return periods of snow water equivalent (SWE or the amount of water stored in the snowpack), which are closely related to potential flooding in spring and water availability in summer, are misrepresented or cannot be reasonably estimated due to a short record length.

Return periods provide insight into the likelihood of the occurrence of a natural phenomenon or hazard (e.g., flood, drought, hurricane, earthquake, tornado) of a given magnitude. They can guide engineering designs and plans. The classic “100-year storm” is an event where the expected time between the occurrences of its magnitude or greater is on average once every 100 years. In other words, such an event has a return period of 100 years or a 1% chance of occurring during any given year. For frequency analysis of annual phenomena (e.g., annual maximum precipitation, SWE, or streamflow), at least a 30-year record is recommended^5^. Various data sources (e.g., satellite, airborne, and ground-based remote sensing, and *in-situ* snow courses and pillows, precipitation gauges, soil moisture sensors, and streamflow gauges) provide information about hydrologic variables, each with their own advantages and disadvantages. This is the case when estimating SWE and other snow characteristics^7^. For instance, satellite remote sensing has led to many advancements in estimating the snowpack’s areal extent, albedo, grain size, depth, and SWE over large, mountainous environments^7–13^; however, the remotely-sensed information does not predate the launch of the relevant satellite. Therefore, the number of observations may be insufficient for statistical analysis. Although some remote sensing products may have adequate record lengths, longer time series may still be desirable for more robust statistical analysis with a larger sample size. On the other hand, many *in-situ* observational networks (e.g., snow courses) can provide measurement records extending back decades before remotely-sensed information was available. However, *in-situ* observations are often point-based and/or spatially-sparse. As we demonstrate herein, fusing multiple data streams together can be used to overcome the mismatch between the number of observations required for robust statistical analysis and the actual amount of data available.

In this study, we focus on merging different sources of SWE information since the seasonal snowpack serves as a critical water reservoir for many regions around the world. It stores precipitation in the winter and releases it as melt runoff in the spring and summer. California, for example, derives one-third of its water from the melting Sierra Nevada snowpack on average^14^, with southern California relying on it for approximately 60% of its water supply^15^. Not only does the snowpack provide vital water resources to people worldwide for agricultural, domestic, municipal, and industrial uses, but snow also supports the multibillion dollar per year global ski industry and tourism^16^ and a variety of ecosystems. We merge SWE volumes from a spatially-distributed, remote sensing-based snow reanalysis with SWE depth measurements from *in-situ* snow courses to extend the SWE volume record for Sierra Nevada, USA. Merging these data streams together, we leverage complementary information from the longer-length *in-situ* measurements and shorter-length reanalysis to estimate Sierra-wide and regional 1 April SWE volumes for nearly 80 years. The resulting 79-year SWE volume records could not have been quantified directly using one of these datasets independently. These derived (data-driven) SWE time series are more than twice as long as existing data-driven distributed SWE time series (e.g., snow reanalyses/reconstructions that rely on remote sensing) that can be used to quantify the SWE volume across this mountain range^2,9,17–19^. As an example application, we perform hydrologic frequency analysis to illustrate the importance of leveraging multiple data streams to generate longer time series for return period estimation. We show the extent to which return periods of the 1 April SWE volume can be misrepresented and how these overestimations/underestimations vary with increasing return periods.

## Methods

### Snow information

**Fig. 1:**
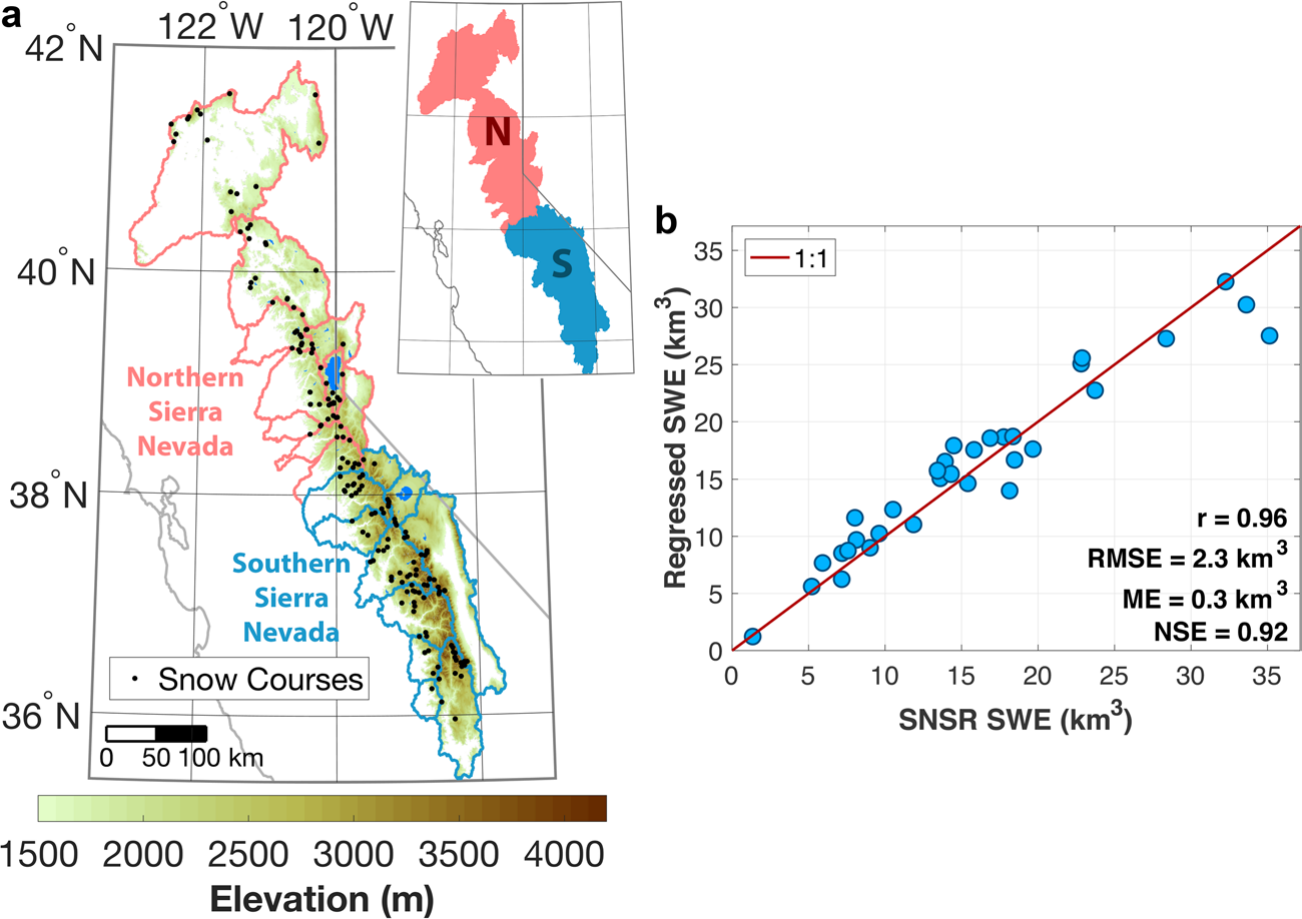
Sierra Nevada domain and verification of regressed 1 April SWE. (**a**) Elevation map from the Sierra Nevada snow reanalysis (SNSR). Shading denotes elevations above 1500 m in the SNSR domain. Locations of snow courses used in this study are demarcated within the 20 delineated river basins. Basins are grouped regionally across the northern (N) and southern (S) Sierra Nevada. (**b**) Sierra-wide regressed SWE from the linear regression model built using snow courses versus SNSR SWE for 1985–2016. The correlation coefficient (r), root-mean-squared error (RMSE), mean error (ME), and Nash-Sutcliffe Efficiency (NSE) are provided.

We use a combination of datasets (a snow reanalysis and *in-situ* snow courses) to derive the long-term SWE volume time series presented and analyzed here. We integrate SWE from the 90-m gridded, daily snow reanalysis^9^ across the Sierra Nevada (Fig. 1a) to compute the mountain range’s integrated SWE volume on 1 April from 1985–2016. Neither measurements from snow courses nor sensors were assimilated during the generation of the Sierra Nevada snow reanalysis (SNSR). Rather, Landsat fractional snow covered area images were assimilated in a Bayesian framework, and the *in-situ* observations were left for independent verification of the resulting SWE fields. Hence, Margulis *et al*.^9^ and Huning & Margulis^20^ highly verified the 1 April SWE and cumulative snowfall from the SNSR with *in-situ* observations from snow courses and sensors. The 32-year SNSR provides only part of the information for the construction of our regression model that also uses snow courses.

To extend the 1 April SWE volume time series beyond the 32 years available directly from the SNSR, we use the average 1 April SWE depth from the California Department of Water Resources (CADWR) snow courses (http://cdec.water.ca.gov/snow/) from 1940–2018. Snow courses tend to be located at low to mid-elevation in relatively flat areas, which may not fully represent the large spatiotemporal heterogeneity of the snowpack or higher elevation SWE across mountainous terrain^17,21–23^. The snow courses used in this study are located at elevations above 1500 m, which is often seasonally snow-covered^24^ and defines the SNSR domain (Fig. 1a). CADWR does not always conduct snow surveys on 1 April, but usually within a few days of the date. Nonetheless, those measurements are considered to be representative of the 1 April snow state. For individual snow courses to be included in the construction of our 79-year SWE volume time series, they must have observations for both 80% of the overlap period with the SNSR and 80% of the entire period of 1940–2018. Figure 1a shows the location of the snow courses utilized. We do not consider snow sensors in this study since they have a shorter record than the courses, and therefore, they would not allow us to substantially increase our temporal window of analysis. We ultimately use the combined information from the SNSR and snow courses to derive the SWE volume over the longer time period, 1940–2018.

Since water managers commonly use 1 April SWE measurements as an indicator of the seasonal snowmelt runoff in the western United States, we focus on this quantity herein. We construct a time series for the entire Sierra Nevada. Since there is high heterogeneity in orographic precipitation and SWE distributions across the mountain range^20,25–29^, resulting from a combination of factors including elevation, land cover, sensitivity and response to warming^21,30^, and differences in storm tracks and characteristics^31–34^, we employ the same methods as described for the Sierra-wide domain for both its northern and southern regions (Fig. 1a).

Table 1 summarizes the sources of information for the generation of the merged SWE volume time series. Below, we describe the construction of the merged datasets using a least squares regression.

**Table 1:** Input SWE information.

Input Data	Source	Link
Sierra Nevada Snow Reanalysis (SNSR)	Margulis *et al*. (2016)	https://ucla.app.box.com/v/SWE-REANALYSIS
Snow Courses	California Department of Water Resources (CADWR)	http://cdec.water.ca.gov/snow/

### Regression and merging data streams

We regress the average SWE depth observed from snow courses and the SNSR SWE volume from 1985–2016 to develop a linear model that maps the average *in-situ* SWE depth to the integrated SWE volume for the mountain range on 1 April. We use this relationship to extend the SWE volume time series to include years 1940–2018. In particular, we construct a merged 79-year dataset, which we call “SWE_RC_” because it uses the reanalysis SWE volume for 1985–2016 (denoted with the subscript “R”) and the SWE volume derived from the regression with snow courses (denoted with the subscript “C”) for 1940–1984 and 2017–2018. We use a similar naming convention for the regional merged datasets– SWE_RC,N_ and SWE_RC,S_ respectively correspond to the merged 1 April SWE volume time series for the northern (N) and southern (S) Sierra Nevada.

Margulis *et al*.^35^ utilized a similar regression-based approach, but leveraged measurements from snow courses to quantify the Sierra-wide peak SWE volumes for water years (1 October-30 September) 1951–2015. Note that the timing of the peak SWE often differs from 1 April. During water years 1985–2016 for instance, the peak SWE for this mountain range occurred from January to May, and on average, it occurred in mid-March^21^. We use a linear regression approach for its simplicity since the Mann-Kendall test^36^ did not detect a trend in the input data or resulting merged dataset at the 0.05 significance level. The p-values from the Mann-Kendall test range from 0.21–0.32 for the SNSR SWE volume (years 1985–2016), 0.13–0.41 for the average SWE depth from the snow courses (1940–2018), and 0.06–0.30 for the merged SWE volume (1940–2018) over the three mountainous domains (northern, southern, and entire Sierra Nevada). Although we do not detect statistically significant SWE trends here, Mote *et al*.^37^ found statistically significant trends in SWE depth from ~35% of the snow courses they examined and ~21% of grid points from a hydrologic model across the western USA. Since we consider SWE aggregated across larger scales, trends occurring at individual sites may not be similarly detected.

Our study verifies that the linear assumption described above provides a reasonable model for building the merged SWE time series over the Sierra-wide, northern, and southern domains. Similarly, if an analysist applies the methods described herein to different regions, variables, etc., the suitability of a linear assumption should also be verified.

### Statistical analysis application

We demonstrate the utility of merging data streams and the extent that capturing additional extreme values can alter the estimation of return periods. We use the Generalized Extreme Value (GEV) distribution to gain a better understanding of the probability of occurrence of the most extreme 1 April SWE volumes across the Sierra Nevada. We fit the GEV distribution using the Processed-informed Nonstationary Extreme Value Analysis (ProNEVA) package^38^ since it provides uncertainty associated with the return level curves through a Markov Chain Monte Carlo (MCMC) approach. Although ProNEVA facilitates both stationary and nonstationary frequency analysis, we use a stationary approach since, as mentioned above, we do not detect a statistically significant trend in the data. As demonstrated below, the appropriateness of a GEV distribution must be determined when fitting a distribution to data for hydrologic frequency analysis.

## Data Records

The merged 1 April SWE volume time series (1940–2018) for the Sierra Nevada domain and the northern and southern regions are publicly available through an online repository^39^. For each domain/region, the dataset is distributed as an ASCII formatted file of the form: Year (column 1) and SWE in km^3^ (column 2).

## Technical Validation

### Sierra-wide performance and uncertainty

A strong relationship emerges between the regressed SWE and SNSR SWE volumes in Fig. 1b during the 32 years of overlap. Summary statistics in Fig. 1b provide information about the performance and uncertainty of the regression model. For instance, the correlation coefficient is only one metric that indicates that exploiting information from the snow courses results in a representative regression model (*r* = 0.96). The regressed SWE has a root-mean-squared error (RSME) of 2.3 km^3^ and is relatively unbiased with a mean error (ME) of 0.3 km^3^ in relation to the SNSR SWE.

We also use the Nash-Sutcliffe Efficiency (NSE)^40^ to further evaluate model performance. NSE values can range from -∞ to unity, where the latter indicates a perfect fit between the regressed SWE and the SNSR SWE, in this case. Models yielding positive NSE values closer to 1.0 are generally taken to exhibit acceptable model performance, whereas values of zero or lower indicate unacceptable model performance where the long-term mean value of the SNSR would provide a better estimate than the proposed regression model^41^. Therefore, the NSE value of 0.92 further supports our use of a simple linear regression and each of the abovementioned performance metrics indicate that this model can reasonably quantify the 1 April SWE volume (relative to the SNSR).

**Fig. 2:**
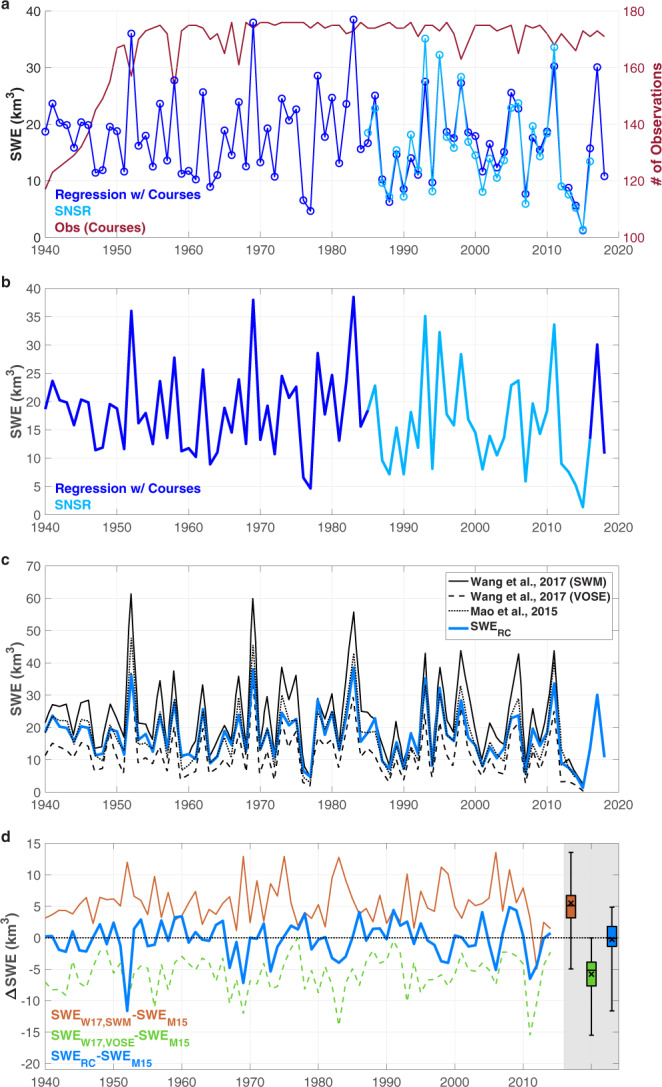
Sierra-wide 1 April SWE volume time series. (**a**) Comparison of SNSR SWE (light blue) and regressed SWE using snow courses (dark blue). The red curve indicates the number of snow course observations. (**b**) Light and dark blue curves combine to form the merged time series, SWE_RC_. (**c**) Comparison of SWE_RC_ to modeled SWE from literature. (**d**) Difference between SWE from datasets in (**c**) and Mao *et al*.^42^ (SWE_M15_). SWE_W17,SWM_ (SWM) and SWE_W17,VOSE_ (VOSE) correspond to SWE values from Wang *et al*.^43^. Boxplots summarize annual differences. ‘ × ’ markers demarcate the mean difference values. Whiskers extend to the most extreme difference values.

Spanning their individual record lengths, Fig. 2a shows both the SNSR (light blue) and regressed (dark blue) SWE volume time series. As demonstrated here, the regressed SWE captures the hydroclimatology of the Sierra Nevada by exhibiting wetter and drier patterns (peaks and troughs) during the same years as the SNSR. From 1940–2018, 117–176 courses (Fig. 2a, red curve) were used annually to generate the regressed SWE. Prior to the 32 years of overlap, fewer snow course observations were available, especially between the 1940s and 1960s. Therefore, greater uncertainty in the SWE time series exists during years with fewer observations and farther away from the period of overlap (i.e., likely earlier in the record).

Figure 2b presents the final, fully-merged 79-year SWE volume time series, SWE_RC_. As shown here, it combines information from the two datasets presented in Fig. 2a (SNSR in light blue and regressed SWE in dark blue). Over the 79 years, the mean SWE volume (and standard deviation) was 17.4 km^3^ (8.1 km^3^). The lowest and highest 1 April SWE occurred in 2015 and 1983, respectively corresponding to ~8 and 222% of the long-term average value.

Lacking long records of SWE volume observations, we compare the SWE_RC_ to modeled SWE derived from a land surface model below to better understand how the data-driven SWE_RC_ performs relative to SWE output from more complex and computationally expensive hydrologic modeling efforts.

### Comparison to SWE from hydrologic modeling

Long-term 1 April SWE datasets for the Sierra Nevada, spanning more than 75 years, have been previously derived using other methods such as land surface modeling (SWE volume^42,43^) and reconstructions with tree rings (SWE depth^44^). Here we focus on the former, since land surface modeling is more commonly used in the hydrologic sciences to provide volumetric SWE estimates. In Fig. 2c, we compare our SWE_RC_ time series to SWE volumes derived by Mao *et al*.^42^ and Wang *et al*.^43^, both of which used the Variable Infiltration Capacity (VIC) macroscale hydrologic model^45^.

As Fig. 2c demonstrates, the 1 April SWE volumes from our SWE_RC_ (blue line) closely agree with the modeled SWE time series from Mao *et al*.^42^ (black dotted line). We estimate the Mao *et al*. SWE from their Fig. 2, which they concluded compares favorably to the Snow Data Assimilation System (SNODAS)^19^ product^42^. Both the SWE_RC_ and Mao *et al*. curves fall within the range of modeled values (solid and dashed black lines) from Wang *et al*.^43^. We estimate the Wang *et al*. SWE from their Fig. S7, where the SWM and VOSE curves correspond to their datasets with the largest and smallest SWE values. Wang *et al*. used five different temperature forcing datasets to illustrate how temperature variability could influence SWE, and thereby increase the uncertainty associated with modeled SWE. Here, the Wang *et al*. curves thereby represent the spread in possible 1 April SWE amounts from models. Of the four datasets presented in Fig. 2c, these two exhibit the lowest (VOSE) and highest (SWM) variance in SWE values from 1940–2014.

The differences between SWE from the SWM, VOSE, and SWE_RC_ time series relative to that from Mao *et al*.^42^ (i.e., the “reference”) are further illustrated in Fig. 2d. SWE_RC_ agrees well with the reference dataset having a slight negative bias. In fact, SWE_RC_ exhibits a mean (median) deviation from the Mao *et al*.^42^ annual SWE values of −0.3 km^3^ (−0.1 km^3^). SWE values from SWM (VOSE) display substantial positive (negative) biases relative to the reference with average deviations of 5.5 km^3^ (−5.8 km^3^). The VOSE dataset is unconditionally negatively biased. There is only one year (2012) in which the 1 April SWE value from SWM is less than the reference.

Overall, our SWE_RC_ dataset compares well with modeled SWE from hydrologic models over the last ~80 years. The approach we use herein is simpler, both in structure and computational effort, than the more complex land surface models, which can require a large number of data inputs (e.g., temperature, wind, precipitation, radiation, soil/vegetation properties, etc.). Since our merged SWE_RC_ dataset integrates SWE across the entire Sierra Nevada to quantify the 1 April SWE volume at the mountain range scale, it does not fully reveal the underlying regional (presented below) or basin scale SWE patterns that can be analyzed using the direct output from the (shorter-length) SNSR or a spatially-distributed hydrologic model. We acknowledge, however, that while distributed hydrologic models provide spatial estimates of SWE, they have their own limitations and sources of errors (e.g., forcing data inputs, model physics parameterizations, etc.). To complement our Sierra-wide data and analysis, we also generate and verify regional 1 April SWE time series for the northern and southern Sierra Nevada below.

### Regional performance and time series

**Fig. 3:**
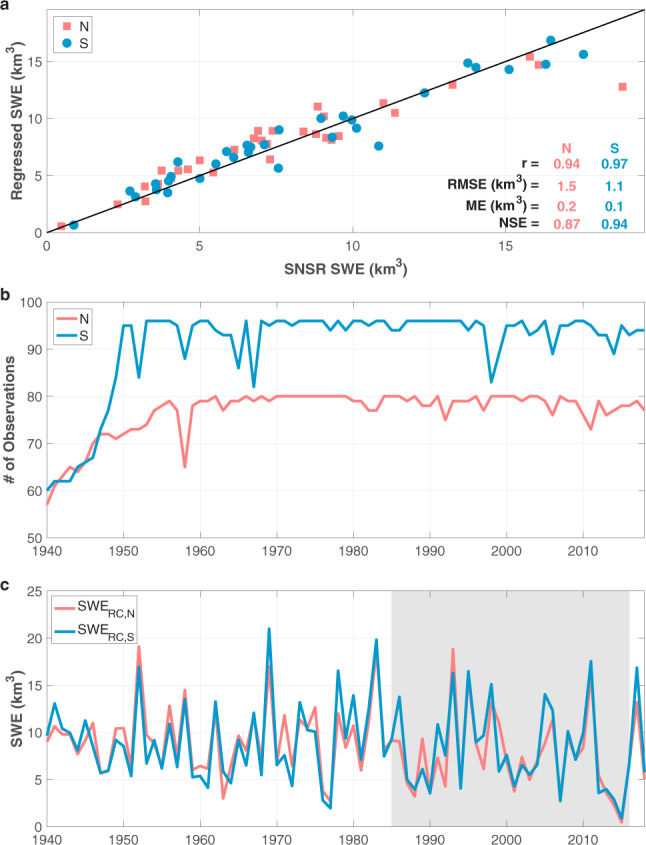
1 April SWE volume verification and time series for the northern (N) and southern (S) Sierra Nevada. (**a**) Regressed SWE from the linear regression model built using snow courses versus SNSR SWE for 1985–2016. Performance metrics (same as in Fig. 1b) are color coded by region. (**b**) Number of snow course observations each year. (**c**) Regional merged time series for the northern (SWE_RC,N_) and southern (SWE_RC,S_) regions.

Here, we present and verify the regional regression-based SWE datasets for the northern and southern Sierra Nevada. Figure 3a indicates good agreement between the regressed and SNSR SWE for the northern (pink) and southern (blue) areas. The performance metrics (r, RMSE, ME, and NSE in Fig. 3a) indicate that the regression model built for the southern Sierra Nevada exhibits better performance than that for the northern region where fewer observations were used to generate the time series (Fig. 3b). Alike the Sierra-wide case, the uncertainty in regional SWE (Fig. 3c) is larger during years with less snow courses and those that are more distant from the overlapping period with regional SNSR SWE. Both the northern and southern merged 1 April SWE time series display similar interannual patterns (Fig. 3c).

While we provide both mountain range scale and regional SWE volume time series, some applications may require further detail at the basin scale. Since not all basins are (equally) sampled with snow courses, or more generally, *in-situ* observations, we focus on SWE volumes over larger areas in this study. The methods described herein may pose useful for creating basin scale datasets where additional spatial resolution or longer-term, merged records are needed; however, in each case, steps must be taken to verify the appropriateness or goodness-of-fit of models/methods used.

## Usage Notes

Given the broad importance of snow to climatic, hydrological, and biogeochemical processes, and the significance of the Sierra Nevada’s 1 April SWE to flood control and water supply in California, we now demonstrate one application of the merged SWE records through hydrologic frequency analysis.

### Sierra-wide hydrologic frequency analysis

**Fig. 4:**
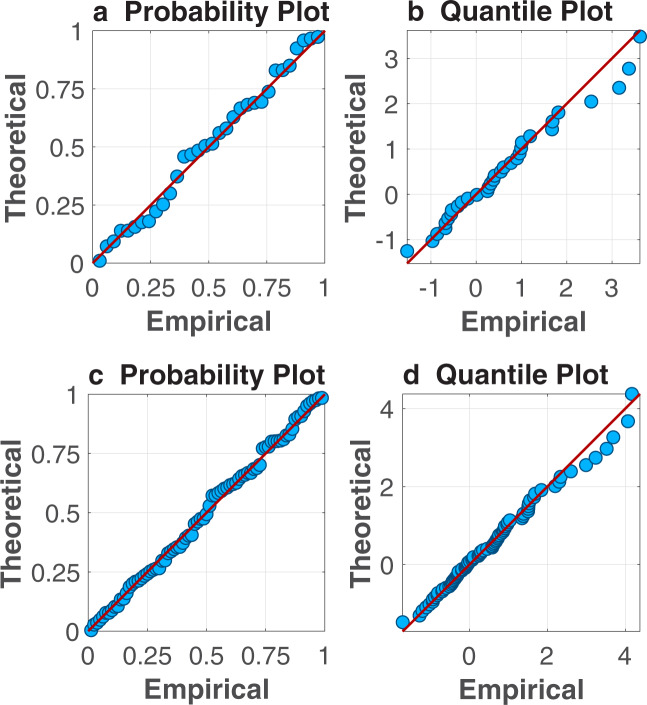
Probability (**a**,**c**) and quantile (**b**,**d**) plots for the Sierra-wide SNSR (**a**,**b**) and SWE_RC_ (**c**,**d**) 1 April SWE volume time series. The theoretical values are derived using the generalized extreme value (GEV) distribution.

**Fig. 5:**
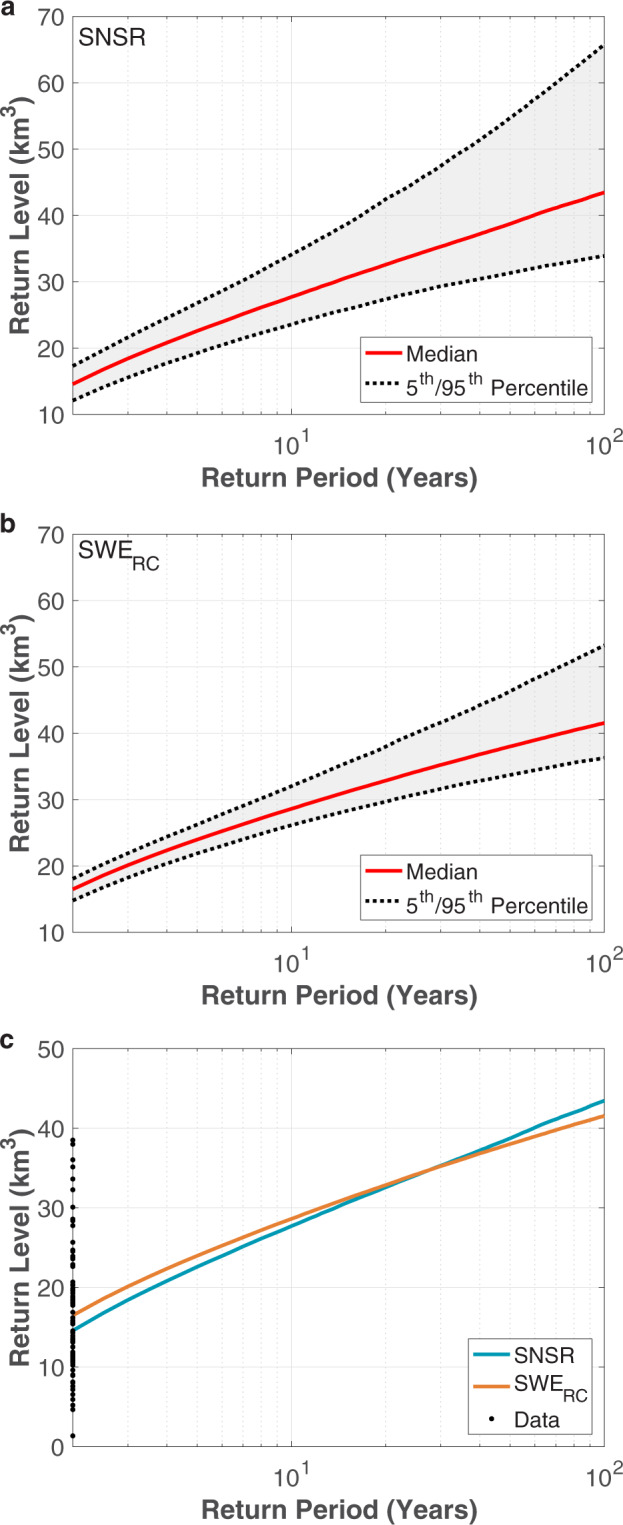
1 April SWE return level curves for the Sierra-wide SNSR (**a**) and SWE_RC_ (**b**) datasets. Solid red and dotted black lines correspond to the median and 5^th^ and 95^th^ percentiles from the Markov Chain Monte Carlo (MCMC) ensemble of simulations. Shading denotes the spread or 90% confidence interval. (**c**) Comparison of the median SNSR and SWE_RC_ return level curves. Dots along the vertical axis denote the 32 SWE volumes from the SNSR time series (i.e., “data”).

**Fig. 6:**
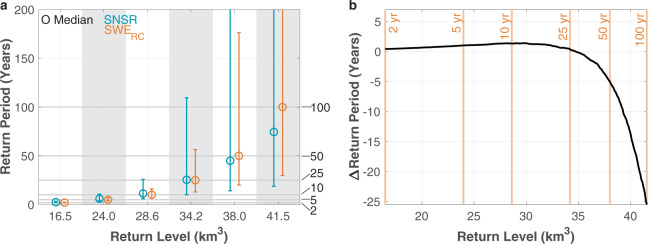
Hydrologic frequency analysis for Sierra-wide 1 April SWE using the SNSR and SWE_RC_. (**a**) Comparison of return periods for specified return levels. Selected return levels are the 1 April SWE values from the SWE_RC_ dataset that have 2-, 5-, 10-, 25-, 50-, and 100-year return periods. Circles demarcate the ensemble median and whiskers correspond to the 5^th^ and 95^th^ percentiles based on the return level curves shown in Fig. 5. The 95^th^ percentiles for the SNSR exceed 200 years for the largest two return levels, whereas this only occurs for the SWE_RC_ in relation to the largest return level. (**b**) Difference between the SNSR and SWE_RC_ return periods (SNSR – SWE_RC_) based on the ensemble median return level curves. Vertical orange lines and values denote the median return periods from the SWE_RC_ that correspond to the same selected return levels as in (**a**). Positive (negative) values correspond to overestimation (underestimation) of the return periods in (**b**) with the SNSR.

The probability and quantile plots in Fig. 4 indicate that the generalized extreme value (GEV) distribution can be used to represent the Sierra-wide SNSR SWE and SWE_RC_ data for frequency analysis. Applying the GEV distribution using ProNEVA^38^, the return level curves for these two datasets are shown in Fig. 5. Figure 6a,b compare the SNSR SWE and SWE_RC_ return periods for specified 1 April SWE amounts or return levels. Relative to the SWE_RC_, the SNSR has greater uncertainty in the return periods associated with a given amount of SWE (Figs. 5 and 6a). In fact, the spread in the return periods for the SNSR is more than two to three times larger than for SWE_RC_ (Fig. 6a) because of the difference in the record lengths.

Now we focus on the ensemble median values as shown in Figs. 5 and 6. For values between 16.5 and 34.7 km^3^, which correspond to return periods of 2 and 27.5 years for the SWE_RC_, the SNSR overestimates the return period by a maximum ~1.4 years. This means that when we use the shorter dataset, it is slightly less likely for those 1 April SWE amounts to be achieved than when estimated with SWE_RC_ (Fig. 6). For perspective, the SWE volume of ~35 km^3^ is comparable to the total capacity of Lake Mead – the largest reservoir in the USA by volume^46^. For return periods larger than 25 years, however, the differences between the two datasets become more pronounced. As an example, when using the SWE_RC_, the 50-year and 100-year 1 April SWE volumes are 38.0 and 41.5 km^3^, respectively. However for these same return levels, the SNSR underestimates the respective return periods by roughly 5 and 25 years. This means that what the short-term record (i.e., SNSR) indicates as the 100-year event is approximately just a 75-year event in the long-term record (i.e., SWE_RC_). Put differently, the short-term record significantly overestimates the frequency (i.e., underestimates the corresponding return period) of extreme SWE conditions (e.g., the 100-yr event) – see Fig. 6. Hence, as the SWE volume increases beyond ~35 km^3^, the point where the difference between return periods from the two datasets is zero, the return periods increasingly diverge for a given amount of SWE. Figure 6 suggests a consistent and substantial underestimation of the return period associated with extremely large SWE amounts when using the shorter SNSR dataset. In other words, the largest 1 April SWE accumulations have larger return periods than suggested by the SNSR, and therefore, the SWE_RC_ indicates that these volumes of SWE are less likely to occur than if the shorter SNSR is used for frequency analysis.

It is worthwhile mentioning that depending on the specific variables considered and temporal periods of analysis, the point where one time series transitions from overestimating to underestimating the return periods (or vice versa) does not always occur. In other words, depending on when extreme values occur, their distribution over time, and their magnitudes, a consistent overestimation or underestimation could occur when comparing return periods from various datasets. Nonetheless, the intersection of the two return level curves in Fig. 5c, and reflected in Fig. 6, should not be unexpected. The curves are derived from datasets differing in length by a factor of more than two (32 versus 79 years) that have distributions with different extreme (and non-extreme) values.

### Regional hydrologic frequency analysis

**Fig. 7:**
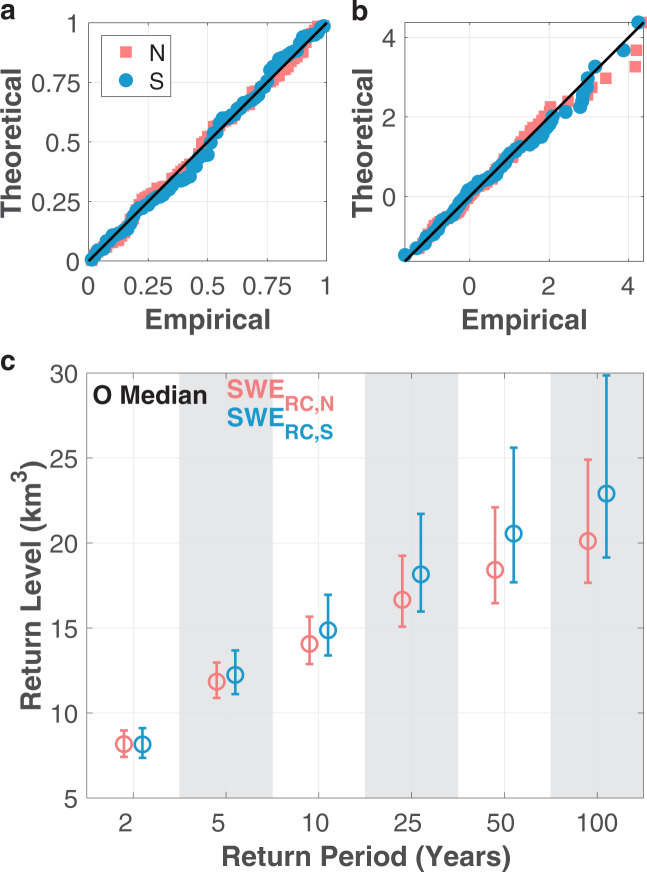
Verification of GEV fit and hydrologic frequency analysis for regional merged 1 April SWE. Probability (**a**) and quantile (**b**) plots for the northern (pink) and southern (blue) SWE volume time series. The theoretical values are derived using the GEV distribution. (**c**) Regional return levels for specified return periods of 2-, 5-, 10-, 25-, 50-, and 100-year. Circles demarcate the ensemble median and whiskers correspond to the 5^th^ and 95^th^ percentiles.

Since the GEV distribution fits the merged SWE_RC,N_ and SWE_RC,S_ well (see Fig. 7a,b), we calculate the SWE volumes corresponding to select return periods (Fig. 7c). By comparing return levels for specified return periods, we provide insight into the likelihood of various amounts of SWE on 1 April in the northern and southern domains. For brevity, we focus on the frequency analysis associated with each of the regional merged datasets, independent of a comparison to the regional SNSR.

The uncertainty associated with both regions increases with increasing return period. The southern Sierra Nevada exhibits larger uncertainty than the northern part (Fig. 7c). For each return period larger than 2 years, the corresponding median return level is larger in the southern portion of the mountain range (Fig. 7c). In fact, the 50-year SWE value of 20.6 km^3^ in the southern area is larger than both the 50-year and 100-year volumes in the northern domain (18.4 and 20.1 km^3^, respectively). The 100-year 1 April SWE volume is therefore also larger in the southern Sierra Nevada with a value of 22.9 km^3^. Overall, larger 1 April SWE volumes are more likely to occur in the southern Sierra Nevada than in the northern region.

Regional data and frequency analysis may provide additional insight that is important for operational use and other applications not possible with only Sierra-wide SWE information. Analysts are encouraged to explore additional applications of the datasets and methods beyond those described in this study. However as noted above, further (spatial) refinement may still be necessary for some analyses (e.g., ecological studies).

In this study, we derive 79-year time series of SWE volumes for the entire Sierra Nevada and the northern and southern parts of this mountain range using a regression-based approach. Performing frequency analysis with the time series, we demonstrate that the shorter Sierra-wide SWE record misrepresents the 100-year 1 April SWE volume by underestimating the return period by roughly 25 years. Since engineering design and planning utilize frequency analysis related to flood control, water supply, and drought mitigation, it is important to understand how data merging techniques can be used to provide new information and/or longer time series for statistical analysis. Figure 6 elucidates how a dataset’s record length and/or the years that it spans can influence return period and risk assessment. Biases in return periods in risk assessment and engineering design and planning applications can substantially alter a population’s level of safety and the costliness of a given project. Robust estimations of return periods and their uncertainty are vital for mitigating natural hazards, safeguarding human well-being, and designing reliable critical infrastructure.

Although we focus on the 1 April SWE given its relevance to reservoirs and flood control, we present a computationally efficient, simple method that could prove valuable for agencies, such as CADWR, when quantifying various hydrologic variables by making use of existing and publicly available long-term *in-situ* records and shorter state-of-the-art remote sensing-related products. We acknowledge that more complicated data merging and fusion techniques exist and they may be required for quantifying other variables or SWE across different locations. Moreover, merging data streams together within a data-driven framework can be more efficient than running complex hydrologic models, which often require a large number of atmospheric and land surface inputs. Overall, our results highlight the strength of combining multiple data streams for hydrologic applications even with a simple regression-based approach.

Given the importance of snow cover to other fields (e.g., climatology, forest and resource management, etc.), our merged datasets should lend themselves to a variety of other applications (e.g., assessing wildfire risk) and also pose new opportunities to better understand hydrologic variability (e.g., the frequency of drought and wet periods) over longer records of time.

## Data Availability

The ProNEVA code is available at http://amir.eng.uci.edu/software.php.
